# Silver Nanoparticles as a Novel Potential Preventive Agent against Acanthamoeba Keratitis

**DOI:** 10.3390/pathogens9050350

**Published:** 2020-05-05

**Authors:** Edyta B. Hendiger, Marcin Padzik, Ines Sifaoui, María Reyes-Batlle, Atteneri López-Arencibia, Aitor Rizo-Liendo, Carlos J. Bethencourt-Estrella, Desirée San Nicolás-Hernández, Olfa Chiboub, Rubén L. Rodríguez-Expósito, Marta Grodzik, Anna Pietruczuk-Padzik, Karolina Stępień, Gabriela Olędzka, Lidia Chomicz, José E. Piñero, Jacob Lorenzo-Morales

**Affiliations:** 1 Instituto Universitario de Enfermedades Tropicales y Salud Pública de Canarias and Departamento de Obstetricia, Ginecología, Pediatría, Medicina Preventiva y Salud Pública, Toxicología, Medicina Legal y Forense y Parasitología, Universidad de La Laguna. Av. Astrofísico Francisco Sánchez S/N, 38203 Tenerife, Spain; edyta.hendiger@wum.edu.pl (E.B.H.); ines.sifaoui@hotmail.com (I.S.); mreyesbatlle@gmail.com (M.R.-B.); atteneri@hotmail.com (A.L.-A.); aitor913@gmail.com (A.R.-L.); carlosbethencourtestrella@gmail.com (C.J.B.-E.); alu0100821895@ull.edu.es (D.S.N.-H.); olfachiboubt@gmail.com (O.C.); ruben_rguez_exposito92@hotmail.com (R.L.R.-E.); jpinero@ull.edu.es (J.E.P.); jmlorenz@ull.edu.es (J.L.-M.); 2 Department of Medical Biology, Medical University of Warsaw, Litewska 14/16, 00-575 Warsaw, Poland; gabriela.oledzka@wum.edu.pl (G.O.); lidia.chomicz@wum.edu.pl (L.C.); 3 Laboratoire Matériaux-Molécules et Applications, La Marsa, University of Carthage, 2070 Carthage, Tunisia; 4 Department of Nanobiotechnology and Experimental Ecology, Institute of Biology, Warsaw University of Life Sciences, 02-787 Warsaw, Poland; marta_grodzik@sggw.pl; 5 Department of Pharmaceutical Microbiology, Centre for Preclinical Research and Technology (CePT), Faculty of Pharmacy, Medical University of Warsaw, Banacha 1B, 02-097 Warsaw, Poland; anna.pietruczuk-padzik@wum.edu.pl (A.P.-P.); karolina.stepien@wum.edu.pl (K.S.)

**Keywords:** *Acanthamoeba*, keratitis, nanoparticles, contact lenses, adhesion

## Abstract

Free living, cosmopolitan amoebae from *Acanthamoeba* genus present a serious risk to human health. As facultative human parasites, these amoebae may cause *Acanthamoeba* keratitis (AK). *Acanthamoeba* keratitis is a severe, vision-threatening corneal infection with non-specific symptoms. The number of reported AK cases worldwide has been increasing every year. Moreover, 90% of *Acanthamoeba* keratitis cases are related to contact lens use. Wearing and storage contact lenses not in accordance with the physicians and manufacturers recommendations are the primary key risk factors of this disease. Amoebae can easily adhere to the contact lens surface and transmit to the corneal epithelium. Preventing amoebae adhesion to the contact lens surface could significantly decrease the number of AK infections. Until now, the effective therapy against AK is still under development. Currently proposed therapies are mainly limited to the chlorhexidine digluconate combined with propamidine isethionate or hexamidine applications, which are insufficient and very toxic to the eye. Due to lack of effective treatment, looking for new potential preventive agents is crucial to decrease the number of *Acanthamoeba* keratitis infections, especially among contact lens users. Nanoparticles have been already included in several novel therapies against bacteria, viruses, fungi, and protist. However, their anti-amoebic potential has not been fully tested yet. The aim of this study was to assess silver nanoparticles (AgNPs) and platinum nanoparticles (PtNPs) anti-amoebic activity and influence on the amoebae adhesion to the surface of four different groups of contact lenses—classified according to the Food and Drugs Administration (FDA) guidelines. The obtained results show that both tested nanoparticles were effective against *Acanthamoeba* trophozoites and decreased the amoebae adhesion to the contact lens surface. AgNPs showed better anti-amoebic activity to cytotoxicity dependence and reduced amoebae adhesion in a wider spectrum of the tested contact lenses. Our studies also confirmed that ionization next to hydration of the contact lens material is a crucial parameter influencing the *Acanthamoeba* adhesion to the contact lens surface. In conclusion, silver nanoparticles might be considered as a novel preventive agent against *Acanthamoeba* keratitis infection.

## 1. Introduction

Free living amoebae (FLA) from *Acanthamoeba* genus have been isolated from both natural and manmade environment sources including sweet and salty water, soil, air, city fountains, and swimming pools. Both *Acanthamoeba* spp. can cause a progressive, sight-threatening corneal infection known as *Acanthamoeba* keratitis (AK). The number of worldwide diagnosed AK cases increases year by year and 90% of them is related to the contact lens use [1,2,3]. Improper management of the contact lenses, washing them in tap water, or wearing them while swimming may provoke contamination with amoebae that can be easily transmitted to the cornea. Amoebae initially localized in the corneal epithelium surface quickly invade the underlying stroma and infiltrate through the corneal nerves, causing neuritis and necrosis [4,5,6,7,8,9]. The infection is commonly one-side and manifests by non-specific symptoms such as severe eye pain, blurred vision, and lachrymation. AK is commonly misdiagnosed with bacterial or viral corneal infections. This mainly results in delay of proper treatment. Consequently, AK can easily lead to blindness [1,10]. Up to date, there is no fully effective therapy available against AK. The therapeutic approaches recommended by the Centers for Disease Control and Prevention (CDC) are chlorhexidine digluconate combined with propamidine isethionate or hexamidine applications. However, the prolonged treatment with these agents is very toxic to the eye and rarely leads to full recovery of the patient [3,11,12,13]. Prevention is still the main factor that limits the number of AK infections.

In recent years, fast development of medical nanotechnology has been observed. Nanoparticles are considered as new potential anti-microbial agents. During this time, their activity has been confirmed against many bacteria, viruses, fungi, and protozoan species [14,15,16,17]. The entire mode of action of nanoparticles is still unknown. Recent studies have revealed that nanoparticles penetrate and disturb the structure of cell membrane, induce intracellular reactive oxygen species (ROS) production, disrupt respiratory chain enzymes, cause cell damage by DNA replication inhibition, affect secondary DNA structure, and inhibit ATP-dependent protein synthesis [18,19]. Silver nanoparticles anti-microbial activity has been described against *Staphylococcus aureus, Klebsiella pneumoniae*, *Pseudomonas aeruginosa*, *Escherichia coli*, *Bacillus subtilis*, *Enterobacter aerogenes*, *Streptococcus mutans*, *Lactobacillus acidophilus*, *Micrococcus luteus,* and *Candida albicans* [20,21,22,23,24,25,26,27]. The anti-protozoal activity of AgNPs has been confirmed against *Echinococcus granulosus*, *Schistosoma japonicum*, *Giardia intestinalis*, *Entamoeba histolytica*, *Cryptosporidium parvum*, *Toxoplasma gondii*, *Leishmania* spp., and *Plasmodium* spp. [14,28,29,30,31,32,33]. The activity of tannic acid-modified silver nanoparticles against *Acanthamoeba* spp. was also confirmed in our recent studies [34]. Platinum nanoparticles have not been as extensively studied as silver nanoparticles but their anti-bacterial activity against *Pseudomonas aeruginosa*, *Bacillus subtilis*, *Listeria monocytogenes*, *Staphylococcus aureus*, *Salmonella enteritidis*, *Candida albicans,* and highly resistant *Escherichia coli* strains has already been described [35,36,37,38,39,40]. Moreover, recent studies have shown that PtNPs may inhibit biofilm formation by *Salmonella typhi* [41]. The anti-protozoal activity of PtNPs has also been investigated and confirmed against *Toxoplasma gondii* [42]. Recent studies using nanotechnologies have focused mostly on the anti-amoebic therapy development. The AK infection prevention improvement using nanoparticles is still an innovative approach that has not been widely tested yet.

Current studies show that the most popular multipurpose contact lens disinfection systems, commonly based on anti-microbial and anti-fungal agents, are not fully effective against *Acanthamoeba*. The amoeba attachment to the contact lens surface is typically the first stage in AK pathogenesis among contact lens users. By reducing the *Acanthamoeba* adhesion, the initiation and progress of the AK infection could be significantly decreased. There is an urgent need to search for a novel, more effective preventive approaches against *Acanthamoeba* spp. infections [43,44,45]. In our previous studies, we confirmed that non-toxic concentrations of silver nanoparticles may significantly enhance the anti-amoebic activity of selected multipurpose contact lens solutions [46]. The positive effect of nanoparticles against contact lens colonization by *Pseudomonas aeruginosa* or *Staphylococcus aureus* has also been observed by other authors [47,48]. In the face of such research results, special attention should be paid to prevent contamination of contact lenses with the *Acanthamoeba* trophozoites and cysts. The adhesion ability of amoebae to contact lens surface is known and influenced by several factors, the most important being water content and ionization. These parameters are used by the FDA to classify hydrogel contact lens materials into four groups. Other factors that may affect the amoebae attachment ability are as follows: silicone content, surface roughness, and contact lens type (soft or hard). Daily or monthly use of contact lenses and their disinfection procedure is also crucial for the possible AK infection. Recent studies have shown that increasing water content and ionization of the contact lens may support adhesion of *Acanthamoeba* trophozoites. Moreover, silicone-containing contact lenses have increased *Acanthamoeba* adhesion compared to other types of contact lenses [49,50,51,52,53].

The aim of this study was to evaluate the AgNPs and PtNPs anti-amoebic activity, cytotoxicity, and potential to reduce amoebae adhesion to the tested contact lens surface in order to confirm their novel preventive potential against *Acanthamoeba* keratitis infection. 

## 2. Results

### 2.1. Characterization of Nanoparticles

**Figure 1:**
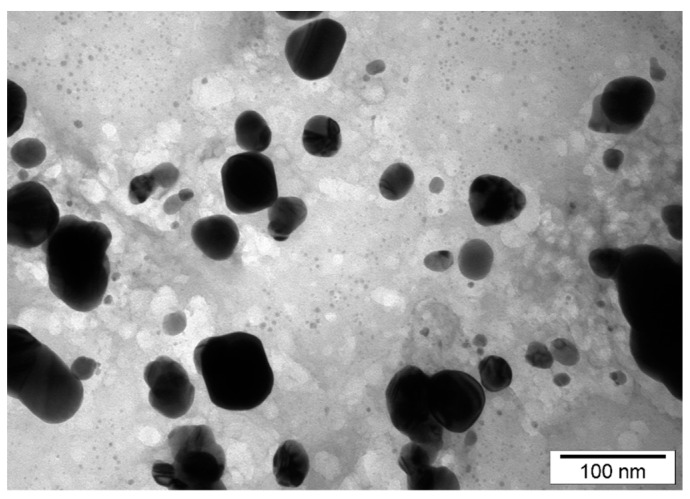
TEM images of silver nanoparticles (AgNPs) distribution and diameter.

**Figure 2:**
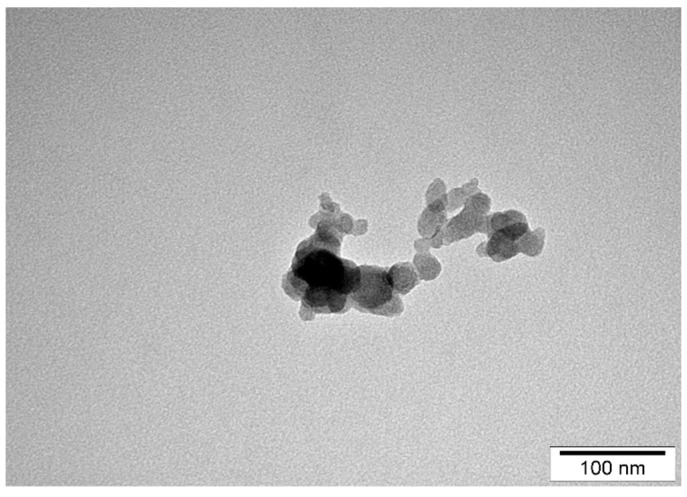
TEM images of platinum nanoparticles (PtNPs) distribution and diameter.

All tested nanoparticles were characterized by Transmission Electron Microscopy (TEM) technique. The size of the AgNPs (Figure 1) ranged from 15 to 30 nm (average value 22.3 ± 12.41 nm). The size of PtNPs (Figure 2) ranged from 4 to 20 nm (average 10.55 ± 7.35 nm).

### 2.2. Activity and Cytotoxicity

**Figure 3:**
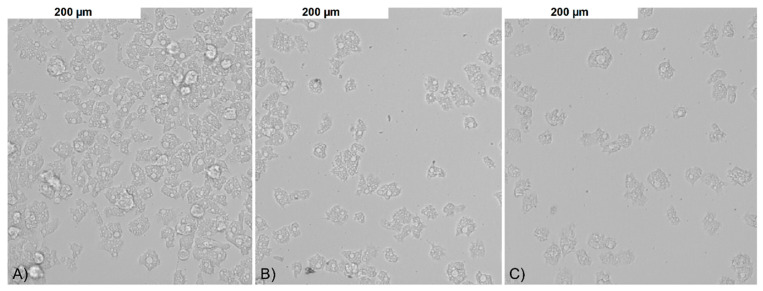
The anti-amoebic activity of the tested nanoparticles after 96 h of incubation: (**A**) control; (**B**) AgNPs 50 ppm; and (**C**) PtNPs 50 ppm.

**Figure 4:**
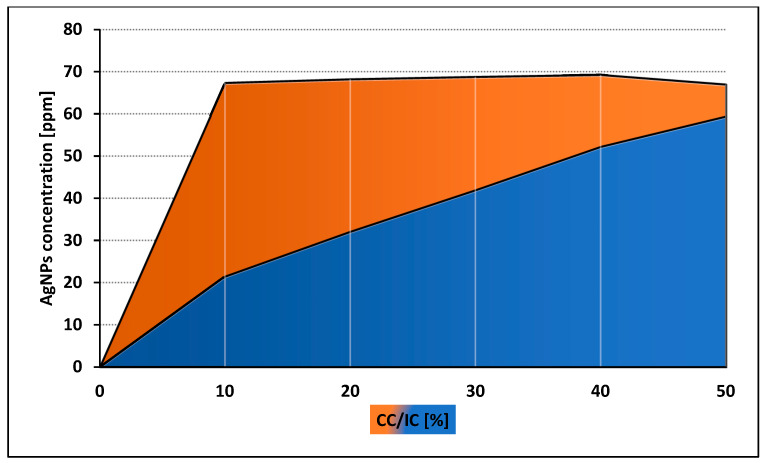
Comparison of anti-amoebic activity (IC) and cytotoxicity (CC) of AgNPs.

**Figure 5:**
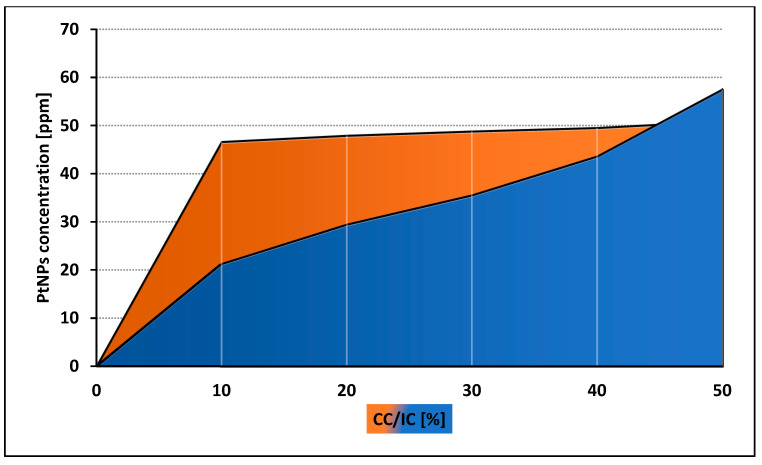
Comparison of anti-amoebic activity (IC) and cytotoxicity (CC) of PtNPs.

Both control and tested assays were visualized to assess number and morphology of the amoebae (Figure 3). The obtained results showed that both tested nanoparticles were active against *Acanthamoeba* Neff strain trophozoites in a dose depended manner. The 50% activity (IC_50_) for AgNPs was 59.42 ± 7.78 ppm and for PtNPs 57.54 ± 5.84 ppm. The measured cytotoxicity of both tested nanoparticles was similar. The calculated 50% cytotoxicity (CC_50_) reached 67.02 ± 4.7 ppm for AgNPs and 61.74 ± 10.08 ppm for PtNPs. In general, the obtained anti-amoebic activity of the AgNPs outweighs their cytotoxicity. In the highest concentrations of PtNPs, the activity to cytotoxicity dependence was negative. The detailed results are presented in Figure 4 and Figure 5.

### 2.3. Adhesion

**Figure 6:**
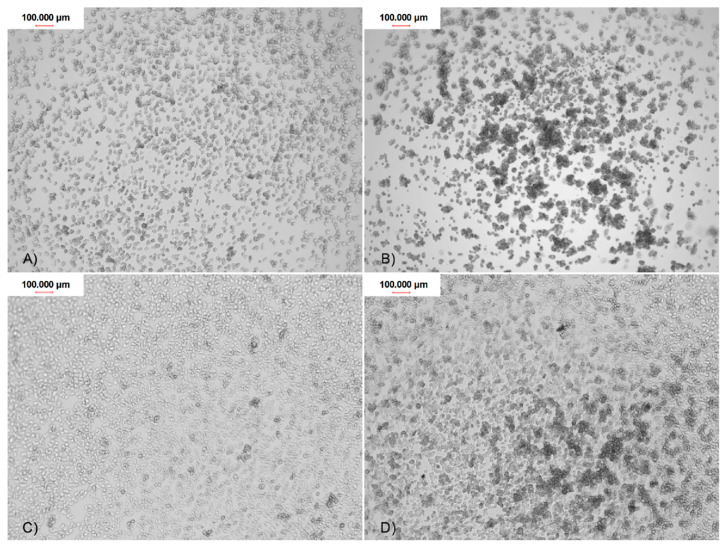
The *Acanthamoeba* trophozoites adhesion to the contact lenses surface of four FDA groups after 90 min of incubation: (**A**) FDA 1; (**B**) FDA 2; (**C**) FDA 3; and (**D**) FDA 4.

The trophozoites adhesion to the contact lenses of FDA 3 and 4 (composed of ionic material) was significantly higher than adhesion to contact lenses of FDA 1 and 2 (composed of non-ionic material). The strong, monolayered adhesion was observed in the FDA 3 and 4 contact lenses. The weakest, irregular adhesion was observed in FDA 1 and 2 (Figure 6). The detailed adhesion observation data are present in Table 1.

**Figure 7:**
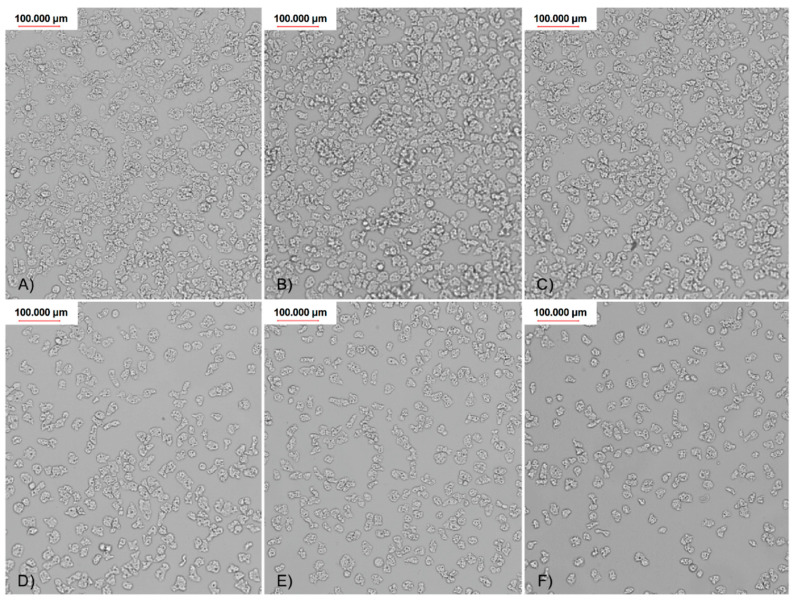
Visualized adhesion reduction on FDA 3 contact lenses, treated with different concentrations of AgNPs: (**A**) control; (**B**) 6,25 ppm; (**C**) 12,5 ppm; (**D**) 25 ppm; (**E**) 50 ppm; and (**F**) 60 ppm.

Generally, all used nanoparticles concentrations influenced amoebae adhesion to all four tested types of contact lenses. The most favorable, dose dependent effect on the adhesion reduction was observed in the FDA 3 contact lenses treated with AgNps (Figure 7). For the other types of contact lenses (FDA 1, 2, and 4), AgNPs were less effective, but still decreased amoebae adhesion to the contact lens surface. In FDA 1 and 2, AgNPs reduced the amoebae adhesion at concentration of 60, 50, and 25 ppm, in the range from 80% to 16%, respectively. At a lower concentration, no anti-adhesive effect was revealed. In FDA 4, the adhesion was reduced residually, only in the highest concentration. PtNPs were generally not as effective as AgNPs. PtNPs significantly reduced the number of adhered amoebae only in the FDA 3 contact lenses and only in the highest concentration used. These nanoparticles did not work in a dose depending manner. In FDA 1, 2, and 4, the anti-adhesive activity of PtNPs was not observed. The counted adhesion reduction (AR) results are presented in Table 2.

## 3. Discussion

*Acanthamoeba* keratitis is a severe corneal infection that can lead to permanent vision loss. The number of patients diagnosed with AK is increasing annually. Up to 90% of the reported cases are recognized among contact lenses users [1,2,3]. The first line of anti-amoebic treatment is mainly limited to diamidines and biguanides applications. These drugs cause alterations in the cytoplasmatic membrane and finally lead to denaturation of cytoplasmatic content of the amoebae cells. Polyhexamethylene biguanide (PHMB) and chlorhexidine are the most often used drugs during AK therapy. In some therapy, chlorhexidine digluconate is combined with diamides such as propamidine isethionate, dibromopropamidine, or hexamidine [2,11,13,54]. Neomycin, as an antibacterial agent, is recommended by some physicians to be included during the initial stage of the therapy. There are also novel therapies being developed. Some of them include using the new generation of antifungals in combination with PHMB, but these were described only in animal models [2]. Summarizing, the available treatment against AK is often prolonged and therefore very toxic to the eye. As the popularity of using contact lenses, as a comfortable vision correction method, is increasing and no definitive effective therapy protocol against AK has been developed yet, the preventive actions including effective contact lens disinfection are crucial to minimize the AK infection rate.

Nanoparticles (NPs), as a new generation of potential anti-microbial agents, have been widely tested against bacteria, viruses, fungi, and protist. Nevertheless, their mechanism of action is not fully understood yet. Due to the small size and surface reactivity, NPs cause cell membrane damages by collapsing the plasmatic membrane potential and subsequently accumulate in cell components. According to the research conducted on bacteria, NPs can inhibit DNA and RNA replication and intracellular enzymes activity. The levels of intercellular ATP levels are depleted and the overall oxidative damages eventually provoke cell death [14,15,16,17,18,19]. The AgNPs, gold nanoparticles (AuNPs), and PtNPs were tested against *Toxoplasma gondii.* The obtained results showed a significant decrease of parasitic growth in non-toxic concentrations of all used NPs. The most favorable effect was described for AgNPs, where IC_50_ achieved less than 1 ppm [42]. The anti-protozoal activity of AgNPs was proved also against *Trypanosoma cruzi*. AgNPs in 100 ppm concentration conjugated with xylene caused 95% of cell death induced by the mechanism of necrosis [55]. The in vitro photodynamic therapy application with using TiO_2_ nanoparticles, doped with Zn, against *Leishmania amazonensis* showed good anti-leishmanial activity and low cytotoxicity to the murine macrophages [56]. The combination of three nanoparticles: silver, chitosan, and curcumin showed promising results in *Giardia lamblia* infection eradication, without toxic effects to the host cells [31]. However, there are only a few studies performed on nanoparticles activity against amphizoic protozoans such as *Acanthamoeba* spp. The previously described study presents that the smallest cobalt phosphate nanoparticles, with the size range 1.30 ± 0.70 µm, showed the best anti-amoebic activity, with only 15% cytotoxicity to HeLa cells [57]. Synthesized TiO_2_ nanoparticles in the concentration of 50 ppm, doped with Zn, reduced by 60% the number of amoebic cells just after 1 h of incubation. The observed positive effect was prolonged up to seven days and acted in a dose depend manner [58]. In other studies, the anti-amoebic effect of chlorhexidine was significantly enhanced by conjugation with 5 μM of AuNPs [59]. At the same time, the toxic effect was reduced from 90% to 40%. Amphotericin B, nystatin, and fluconazole also showed the increase of anti-amoebic activity after conjugation with AuNPs [60]. In our previous studies, we showed a lack of AuNPs activity against *Acanthamoeba* trophozoites [34]. Up to date, no studies are describing the influence of PtNPs against *Acanthamoeba*. AgNPs are currently the best studied nanoparticles in terms of their anti-amoebic activity. Recent studies showed that AgNPs, after 96 h of incubation, may decrease both metabolic activity and adherence ability of *Acanthamoeba* [61]. In the current study, we showed that amoebae adherence to the contact lenses was significantly decreased just after 6 h of incubation. In our previous studies, adsorption and internalization of silver nanoparticles by the *Acanthamoeba* trophozoites were confirmed by TEM techniques [34]. It was confirmed that AgNPs enhanced the bioactivity of amphotericin B and nystatin [62]. Recent publications also propose the application of anti-diabetic drugs such as Glimepiride, Vildagliptin, and Repaglinide together with AgNPs as a novel approach to the AK therapy. It was confirmed that low concentrations of Vildagliptin coated with AgNPs showed good anti-amoebic effect and additionally inhibited the encystation [63].

Contact lens types are classified according to varied factors including hydration and ionic properties of the material, silicone presence, softness, hardness or using period and dedication. FDA classifies contact lenses depending on the material from which they are made into four groups based on the water content and ionization. FDA 1 and 2 represent non-ionic materials with low (<50%) and high (>50%) water content, respectively. FDA 3 and 4 represent ionic materials with low (<50%) and high (>50%) water content, respectively. The *Acanthamoeba* adhesion to contact lens surface is a key first step in the pathogenesis of AK. Adhered *Acanthamoeba,* by the presence of minor corneal abrasions, can easily be transmitted from contact lens surface to the corneal epithelium. It is known that *Acanthamoeba* adhesion to the contact lenses varies depending on the contact lens material [1,4,49,50,53]. The adherence ability of *Acanthamoeba* was described in previous studies where eight types of contact lenses belonging to four FDA groups were exposed to *Acanthamoeba castellanii* and *Acanthamoeba polyphaga* trophozoites and cysts [64]. The adhesion was observed after 90 min of incubation. The parameters that influenced increased *Acanthamoeba* adherence were ionization and high-water content of the material. Other studies confirmed that only contact lens hydration significantly increased the amoebae adhesion level [53]. Our results confirm that both water content and ionization increased *Acanthamoeba* adhesion to the contact lens surface. The highest adhesion was observed on FDA 3 and 4 contact lenses, which both represent ionic materials but different water content— 36% and 55% of hydration, respectively. In FDA 1 and 2 contact lenses, which are made of non-ionic materials, the observed adhesion was significantly lower and irregular. The presence of silicone in the contact lens material, next to hydration and ionization, also plays an important role in the *Acanthamoeba* adhesion. In the studies performed by Lee at al. and Omaña-Molina et al., three generations of silicone hydrogel materials were tested. Lotrafilcon A and B, Senofilcon A, Galyfilcon A and Comfilcon A showed high *Acanthamoeba* adhesion [52,65]. In our studies, we also confirmed that content of silicone may increase the *Acanthamoeba* adhesion. However, very high adhesion was also observed in Methafilcon A (FDA 4), which is an ionic material with high water content and no silicone presence. We conclude that ionization might be a more important parameter that influences the *Acanthamoeba* adhesion than hydration and silicone presence. It is also worth noting that FDA 3 materials are mostly used in monthly contact lenses. This may significantly increase the risk of AK. It was proved that contamination of the contact lenses by bacterial biofilm enhanced the surface adhesion of *Acanthamoeba* [49,66].

Current contact lenses disinfection systems include mostly anti-bacterial agents such as polyhexamethylene biguanide (PHMB) and polyquaternium-1 (PQ-1) [43,44,45,46]. Three popular contact lens care solutions, namely Solo Care Aqua, Renu Multiplus, and Opti Free, were examined. Their anti-microbial activity against *Pseudomonas aeruginosa* and *Staphylococcus aureus* was confirmed. However, anti-amoebic activity against *Acanthamoeba castellanii* and *Acanthamoeba polyphaga* was not sufficient [67]. Other reports show a lack of anti-amoebic activity in five commercially available contact lens solutions [68]. In our previous studies, we also confirmed the lack of amoebicidal activity of the most popular contact lens disinfection systems [46]. The contamination of contact lens solutions samples, obtained from patients during 2009–2014, with *Acanthamoeba* spp. achieved 8.6%. In some cases, samples were co-cultured with bacteria and fungi [69]. Recent studies have shown promising results of contact lens care solutions with autophagy inhibitors combination on amoebae adhesion. However, the time of incubation used in this study was 18 h which is three times longer than the minimum disinfection time recommended by the contact lens solutions manufacturers [70]. In the current study, we showed that 60 ppm of AgNPs reduced *Acanthamoeba* adhesion to the contact lens surface over 50% in just 6 h of incubation. The obtained anti-adhesive effect was dose dependent. Moreover, our previous studies confirmed that conjugation of selected contact lens solutions with silver nanoparticles may significantly increase their anti-amoebic activity without increased cytotoxic effect against murine fibroblasts [46]. The other studies showed that coating contact lenses with AgNPs can reduce *Pseudomonas aeruginosa* adhesion [48]. On the other hand, PtNPs reduced significantly *Acanthamoeba* adhesion only in the highest and most cytotoxic concentration. Moreover, anti-adhesive effect was revealed only for FDA 3 contact lenses. Laboratory synthesis of the PtNPs is also more expensive than synthesis of the same amount of AgNPs. Considering the aforementioned factors, AgNPs seem to be more promising as a novel potential preventive agent against *Acanthamoeba* keratitis.

## 4. Materials and Methods

### 4.1. Cultivation of the Strain

*Acanthamoeba castellani* NEFF (ATCC 30010) type strain of the American Type Culture Collection (LG Promochem, Barcelona, Spain) was used to test the anti-amoebic activity of the nanoparticles. The strain was axenically cultured in culture tissue flasks at 27 °C in Peptone Yeast Glucose (PYG) medium (0.75% (*w/v*) protease peptone, 0.75% (*w/v*) yeast extract, and 1.5% (*w/v*) glucose) containing 10 mg of gentamicin mL^−1^ (Biochrom AG, Cultek, Granollers, Barcelona, Spain) at the Institute of Tropical Diseases and Public Health, University of La Laguna, Spain. The strain was subcultured three days before tests were performed and observed for their growth under a Leica DM IL Invert microscope.

For the toxicity assays, the murine macrophages J774A.1 (ATCC # TIB-67) cell line was cultured in Dulbecco´s Modified Eagle´s medium (DMEM, *w/v*) supplemented with 10% (*v/v*) fetal bovine serum with 10 µg/mL gentamicin (Sigma-Aldrich, Madrid, Spain), at 37 °C and 5% CO_2_ atmosphere.

For experiments performing, all strains were used in the logarithmic phase of growth.

### 4.2. Nanoparticles

Both nanoparticles used in this study were kindly provided by the Department of Nanobiotechnology and Experimental Ecology, Institute of Biology, Warsaw University of Life Sciences, Poland. The stocks solutions in concentrations 100 ppm for the experiments were prepared in water and maintained at 27 °C in darkness until required for the experiments. Stock solutions were sonicated before tests performing.

AgNPs were purchased from Sigma Aldrich, UK (Cat. No. 576832). PtNPs were obtained from Nano-Koloid sp. z o.o (Poland). The distribution and size of nanoparticles were inspected by transmission electron microscopy (TEM) using a JEOL JEM-1220 TE microscope at 80 KeV (JEOL Ltd., Japan), with Morada 11-megapixel Camera (Olympus Corporation, Japan). The Zeta potential of the nanoparticles was measured by electrophoretic light-scattering method, using Zetasizer Nano-ZS90 (Malvern, Worcestershire, UK). Each sample was measured after 120 s of stabilization at 25 °C, pH 8.6, in 20 replicates.

### 4.3. Activity Assays

PtNPs and AgNPs at concentrations of 100, 50, 25, 12.5, 6.25, and 3.125 ppm were examined and compared for their anti-amoebic activity.

The activity of the tested nanoparticles against the trophozoite stage of *Acanthamoeba castellani* NEFF was determined in vitro using a previously described, 96-well microtitrate plate colorimetric assay based on the assay oxide-reduction of alamarBlue® reagent (Life Technologies, Barcelona, Spain).

Briefly, the trophozoites were counted using a Countess II FL automatic cell counter (Thermo Fisher Scientific, Madrid, Spain) to prepare a working cell suspension (10^4^ cells/well) and 50 µL per well were seeded in a 96-well plate (Thermo Fisher Scientific, Madrid, Spain). Amoebae were allowed to adhere to the bottom of the microtitrate plate well.

During amoebae adhering, a serial dilution of the PtNPs and AgNPs in the deep well plate was prepared. Nanoparticles were diluted in the same culture medium (PYG). After that, 50 μL of the serial dilution series of nanoparticles solution were added. As a negative control, *Acanthamoeba* trophozoites were incubated only with the medium. Finally, the alamarBlue® reagent was positioned into each well (the amount equal to 10% of medium volume) and the plate was incubated in 28 °C with slight agitation. Subsequently, the emitted fluorescence was analyzed over a period of 96 h with an EnSpire® Multimode Plate Reader (Perkin Elmer, Madrid, Spain) using a wavelength of 570 nm and a reference wavelength of 630 nm. To calculate the percentages of growth inhibition and 50% inhibitory concentrations (IC_50_), a nonlinear regression analysis was performed with a 95% confidence limit using the SigmaPlot 12.0 software (Systat Software Inc., London, UK). The inhibition curves of the analysis were developed. All experiments were performed in triplicate and the standard division and mean values were also calculated. A paired two-tailed *t*-test was used for the analysis of the data and the values of *P* < 0.05 were considered statistically significant. The ICs of the compounds were calculated using a GraphPad calculator (GraphPad Software, San Diego, CA, USA) based on the calculated IC_50_ and the Hill Slope (H). Amoebae in both control and tested assays were visualized by Microscope Evos fl Cell Imaging System.

### 4.4. Cytotoxicity Assays

To evaluate and compare the cytotoxicity of the used nanoparticles, the murine macrophage cell line J774A.1 (ATCC TIB-67) was used. The murine macrophages are more robust and stable in a laboratory conditions than human corneal cells and therefore provide more accurate cytotoxicity results [3]. Firstly, the macrophages were cultured in RPMI 1640 medium without phenol red (Roswell Park Memorial Institute, Thermo Fisher Scientific Inc., Waltham, MA, USA). Simply, J774A.1 cells were plated in 96-well plates (10^5^ cells per well). After that, the cells were seeded (50 µL) in 96-well plates and serial dilutions (diluted in medium) of the nanoparticles were added (50 µL) to achieve a final volume of 100 µL per well, as previously described. As negative control, macrophages incubated only with the medium were used. Finally, the alamarBlue® reagent was placed into each well (10% of final volume) and incubated for 24 h at 37 °C and 5% CO_2_ atmosphere.

The plates were analyzed with an EnSpire® Multimode Plate Reader as mentioned above. To calculate the 50% value of cytotoxicity (CC_50_), the statistical analysis software SigmaPlot 12.0 was used as previously reported. All experiments were performed three times and the mean values and standard division were calculated. The CCs of the compounds were calculated using a GraphPad calculator (GraphPad Software, San Diego, CA, USA) based on the calculated CC_50_ and the Hill Slope (H). Macrophages in both control and tested assays were visualized by Microscope Evos fl Cell Imaging System.

### 4.5. Adhesion Assays

To evaluate influence of selected nanoparticles on the *Acanthamoeba* Neff strain adhesion ability, tests using hydrogel contact lenses (Table 3) belonging to FDA 4 were performed. Each contact lens was placed inside the 24-well microtitrate plate and exposed to 1000 µL of the *Acanthamoeba* culture at concentration of 10^5^ cells per well. After 90 min of incubation at room temperature, each contact lens coated by the amoebae was carried to another well, washed with PYG medium, and monitored for the number of attached trophozoites. After that, coated contact lenses were exposed to 1000 µL of nanoparticles at concentrations of 60, 50, 25, 12.5, and 6.25 ppm and PYG medium for the control wells. Plates were incubated 6 h at room temperature. After that, contact lenses were transmitted to fresh wells, washed with PYG medium, and counted for number of adhered amoebae. The experiments were performed three times and the Amoebae were visualized by inverted microscope OPTA-TECH MW50 with OPTA-TECH MI5FL 5MPix digital camera. Adhesion reduction (AR) was calculated using below formula,

(1) $$\mathrm{AR}=\frac{\left(\mathrm{nc}-\mathrm{nt}\right)}{\mathrm{nc}}\times 100\%$$

where nc is number of attached amoebae in the control well and nt is number of attached amoebae in the test well.

## 5. Conclusions

The results of our studies reveal that both tested types of nanoparticles showed anti-amoebic activity against *Acanthamoeba* trophozoites. Moreover, silver nanoparticles decreased significantly *Acanthamoeba* adhesion to the surface of all tested four types of contact lenses. The most favorable, dose-dependent effect of AgNPs at non-toxic concentration was observed on FDA 3 contact lenses. Contrarily, platinum nanoparticles significantly reduced number of adhered amoebae only on FDA 3 contact lenses. Our studies also confirmed that ionization next to hydration of the contact lens material is a crucial parameter influencing the *Acanthamoeba* adhesion to the contact lens surface. In accordance with the obtained results, AgNPs seem to be more promising as a novel potential preventive agent against *Acanthamoeba* keratitis. Therefore, further studies are planned on the cysts stage and to evaluate the influence of non-toxic AgNPs concentrations conjugated with contact lens solutions on *Acanthamoeba* adhesion to the selected contact lenses.

## Figures and Tables

**Table 1:** The detailed *Acanthamoeba* adhesion observation data.

	FDA 1	FDA 2	FDA 3	FDA 4
adhesion arrangement	not regular	not regular, grouped	monolayer	monolayer
adhesion strength	mild	mild	strong	strong
detachment of cells after washing	moderate	strong	mild	mild

**Table 2:** Adhesion reduction (%) results after treatment with different concentrations of nanoparticles in the FDA 3 contact lenses.

NPs Concentration (ppm)	AgNPs	PtNPs
60	55.46	43.51
50	42.92	12.98
25	48.38	2.21
12.5	10.18	0
6.25	0	0

**Table 3:** Characterization of the FDA types of hydrogel contact lenses used in the study.

Polymer	FDA Group	Water Content	Ionic	Silicon Content	Manufacturer
Senofilcon A	1	38%	no	yes	ACUVUE oasys
Hilafilcon B	2	59%	no	no	Baush&Lomb SofLens
Balafilcon A	3	36%	yes	yes	Baush&Lomb PureVision
Methafilcon A	4	55%	yes	no	FitView
